# *Bipolaris* or *Curvularia*? Resolving the spicy issue of how clinical isolates should be reported

**DOI:** 10.1371/journal.ppat.1012678

**Published:** 2024-11-20

**Authors:** Sarah E. Kidd, Lars F. Westblade

**Affiliations:** 1 National Mycology Reference Centre, Microbiology & Infectious Diseases, SA Pathology, Adelaide, South Australia, Australia; 2 School of Biological Sciences, Faculty of Sciences, University of Adelaide, Adelaide, South Australia, Australia; 3 Departments of Pathology and Laboratory Medicine, Medicine, and Pediatrics, Weill Cornell Medicine, New York, New York, United States of America; Vallabhbhai Patel Chest Institute, INDIA

## Taxonomic changes in *Bipolaris* and *Curvularia* affect clinical isolates

Over the past 2 decades, mycology has made an important leap towards incorporating molecular phylogeny into taxonomy and classification. *Bipolaris* and *Curvularia* species are dematiaceous moulds that have undergone significant taxonomic reclassification on the basis that neither genus was monophyletic and conidial morphology is not a reliable characteristic to differentiate these genera [1–3]. As a result, the majority of clinically important *Bipolaris* species have been transferred to *Curvularia*; in particular, *B*. *australiensis*, *B*. *hawaiiensis*, and *B*. *spicifera* are now *Curvularia* species (*C*. *australiensis*, *C*. *hawaiiensis*, and *C*. *spicifera*) and collectively make up the “*spicifera* clade” [3].

## *Bipolaris* taxonomic changes present a reporting conundrum for clinical microbiology laboratories

While *Bipolaris* and *Curvularia* species have traditionally been differentiated based on conidial characteristics, most clinical microbiology laboratories lack the capacity to identify these isolates to the species level. Thus, without being able to differentiate *Curvularia spicifera* (formerly *Bipolaris spicifera*) from *Bipolaris cynodontis*, for example, based on morphology, it may be unclear how an isolate with the morphological features of a traditional small-conidium *Bipolaris* species (that is, having straight conidia with pseudoseptae) should be reported to clinicians. That is, should these isolates be reported as *Bipolaris* species or *Curvularia* species? And does this have clinical implications? In this Pearl, we address these questions and, importantly, provide reporting guidance for clinical microbiology laboratories.

## *Curvularia* (*Bipolaris*) *“spicifera* clade” members dominate published human case reports

To determine those *Bipolaris* species recovered from human clinical specimens reported in the literature, we accessed case reports through PubMed (https://pubmed.ncbi.nlm.nih.gov/) using the following search terms: “*Bipolaris*,” “case,” "human,” and “report” (https://pubmed.ncbi.nlm.nih.gov/?term=Bipolaris+case+human+report [accessed 2024 March 6]). The search yielded 96 reports published between 1986 and 2023. Of these, 18 were excluded for various reasons (for example, review articles rather than case reports, not relating to fungal infection, and non-English language reports) leaving 78 for analysis. Of the remaining reports, 79% (62/78) reported the organism to the species level. The overwhelming majority of these case reports (94% [58/62]) identified members of the “*spicifera* clade” (*B*. *australiensis* [7 reports]; *B*. *hawaiiensis* [21 reports]; *B*. *spicifera* [29 reports]; *B*. *hawaiiensis* and *B*. *spicifera* [1 case series report]), revealing the predilection of “*spicifera* clade” members for human infection.

## *Curvularia* (*Bipolaris*) *“spicifera* clade” members dominate isolates recovered from clinical specimens

A review of archived human clinical isolates handled in the National Mycology Reference Centre (Australia) that were reported as “*Bipolaris* species” between 2016 and 2024 revealed 23 isolates. Isolates were recovered from a wide range of specimens, including nasal and sinus specimens, respiratory aspirates, subcutaneous tissue and wound specimens, and corneal tissue. All isolates were recultured and reidentified by sequencing of the internal transcribed spacer (ITS) ribosomal DNA and glyceraldehyde-3-phosphate dehydrogenase (GAPDH) gene [1], with sequence comparisons to the Mycobank and NCBI databases using the BLASTn algorithm (https://www.mycobank.org/page/Pairwise_alignment; https://blast.ncbi.nlm.nih.gov/Blast.cgi). Of the 23 isolates, 91% (21/23) were identified as *Curvularia* species (*C*. *hawaiiensis* [4 isolates], *C*. *lunata* [2 isolates], *C*. *paterae* [1 isolate], *C*. *spicifera* [13 isolates], and *C*. *tribuli* [1 isolate]); 74% (17/23) of these isolates are considered part of the “*spicifera* clade.” The 2 *C*. *lunata* isolates were originally misidentified as *Bipolaris* species, having conidia that were observed to lack the characteristic enlarged subterminal cell, thus more closely resembling conidia of a traditional *Bipolaris*. This highlights the fallibility of conidial morphology in differentiating traditional *Bipolaris* and *Curvularia* genera. Two isolates were confirmed as *Bipolaris* species (1 isolate each of *B*. *cynodontis* and *B*. *yamadae*). The species diversity observed among these Australian clinical isolates is similar to that observed in a study of isolates from the United States [4].

## *Curvularia* (*Bipolaris*) “*spicifera* clade” members and other *Curvularia* species have comparable antifungal susceptibilities

A valid concern related to the reporting of “*spicifera* clade” species as *Curvularia* is that important therapeutic considerations could be overlooked, and thus patient management may be compromised by reporting all clinical *Bipolaris* isolates as “*Curvularia* species.” To investigate this possibility, we compared antifungal susceptibility testing data for “*spicifera* clade” species to other *Curvularia* species (*C*. *brachyspora*, *C*. *geniculata*, *C*. *lunata*, and *C*. *pallescens*). Minimum inhibitory concentration (MIC) value distributions for amphotericin B, itraconazole, posaconazole, and voriconazole are tabulated in Table 1. MIC values for *Bipolaris* (*sensu stricto*) species were not available for comparison, highlighting the relative paucity of these species in clinical specimens and their involvement in human infection. Most modal MIC values for the “*spicifera* clade” were the same as those of other clinically important *Curvularia* species or differed by only 1 doubling dilution. The majority of modal MIC values for the *C*. *spicifera* clade versus other *Curvularia* species are within 1 doubling dilution. Further, the MIC_50_ and MIC_90_ values for both groups were identical or within 1 doubling dilution, with the exception of the itraconazole MIC_90_ value (*C*. *spicifera* clade 0.5 μg/mL; other *Curvularia* species, 8 μg/mL). The greater than 1 doubling dilution difference observed for the itraconazole MIC_90_ values is a result of 9 (14% [9/65]) non-*spicifera* clade isolates exhibiting an itraconazole MIC value of 8 μg/mL. Overall, these data indicate antifungal MIC values for *C*. *spicifera* clade members are largely comparable to other *Curvularia* species.

**Table 1:** Distribution of minimum inhibitory concentration (MIC) values of *C*. *spicifera* clade members and other *Curvularia* species tested in the National Mycology Reference Centre (Australia) according to the M38M51S Clinical and Laboratory Standards Institute (CLSI) broth microdilution reference method [5].

Antifungal agent	Genus/clade	No. isolates	MIC value (μg/mL)										Modal MIC	MIC_50_	MIC_90_
			0.016	0.03	0.06	0.125	0.25	0.5	1	2	4	8			
Amphotericin B	*C*. *spicifera* clade^a^	36		1		7	8	7	11	2			1	0.5	1
	Other *Curvularia* species^b^	65			2	3	12	13	20	11	4		1	1	2
Itraconazole	*C*. *spicifera* clade^a^	36		1	4	6	16	8	1				0.25	0.25	0.5
	Other *Curvularia* species^b^	65			4	17	28	5	1	1		9	0.25	0.25	8
Posaconazole	*C*. *spicifera* clade^a^	32	2	4	5	8	7	4		2			0.125	0.125	0.5
	Other *Curvularia* species^b^	60	1	5	8	18	20	3	5				0.25	0.125	0.5
Voriconazole	*C*. *spicifera* clade^a^	35				4	9	9	5	8			0.25, 0.5	0.5	2
	Other *Curvularia* species^b^	64			2	5	13	16	17	5	6		1	0.5	2

^a^Includes *C*. *australiensis*, *C*. *hawaiiensis*, and *C*. *spicifera*, formerly classified within *Bipolaris*.

^b^Includes *C*. *brachyspora*, *C*. *geniculata*, *C*. *lunata*, and *C*. *pallescens*.

## Support for reporting all clinical *Bipolaris* and *Curvularia* isolates as “*Curvularia* species”

An overwhelming majority of published *Bipolaris* case reports indicate a causative organism of human infection that is now classified within the genus *Curvularia*, and similarly, reidentification of clinical isolates that were reported as “*Bipolaris* species” demonstrates that most are now considered to be *Curvularia* species. Furthermore, there are minimal differences in antifungal MIC distribution values observed between *C*. *spicifera* clade species (former *Bipolaris* species) and other traditional *Curvularia* species. Taken together these findings provide justification for clinical microbiology laboratories to report all clinical isolates recovered from human specimens that morphologically resemble either *Bipolaris* or *Curvularia* simply as “*Curvularia* species.” Genus-level identification of clinically important moulds, at least in the first instance, is often sufficient for effective clinical management. Of course, isolates can be referred for definitive identification where there is a need, such as in the case of unusual morphology, or as would be required in the setting of an outbreak for epidemiological purposes. Finally, to emphasise its importance and to assist in recalling this important taxonomic and nomenclatural change, we conclude this Pearl with a haiku.

*Bipolaris mourns*

*spicifera is transformed*

*Curvularia*
